# High Frequency of *AIFM1* Variants and Phenotype Progression of Auditory Neuropathy in a Chinese Population

**DOI:** 10.1155/2020/5625768

**Published:** 2020-07-01

**Authors:** Hongyang Wang, Dan Bing, Jin Li, Linyi Xie, Fen Xiong, Lan Lan, Dayong Wang, Jing Guan, Qiuju Wang

**Affiliations:** ^1^College of Otolaryngology, Head and Neck Surgery, Chinese PLA Institute of Otolaryngology, Chinese PLA General Hospital, Beijing 100853, China; ^2^National Clinical Research Center for Otolaryngologic Diseases, Beijing 100853, China; ^3^Department of Otolaryngology-Head and Neck Surgery, Tongji Hospital, Tongji Medical College, Huazhong University of Science and Technology, Wuhan 430030, China

## Abstract

To decipher the genotype-phenotype correlation of auditory neuropathy (AN) caused by *AIFM1* variations, as well as the phenotype progression of these patients, exploring the potential molecular pathogenic mechanism of AN. A total of 36 families of individuals with AN (50 cases) with *AIFM1* variations were recruited and identified by Sanger sequencing or next-generation sequencing; the participants included 30 patients from 16 reported families and 20 new cases. We found that *AIFM1*-positive cases accounted for 18.6% of late-onset AN cases. Of the 50 AN patients with *AIFM1* variants, 45 were male and 5 were female. The hotspot variation of this gene was p.Leu344Phe, accounting for 36.1%. A total of 19 *AIFM1* variants were reported in this study, including 7 novel ones. A follow-up study was performed on 30 previously reported *AIFM1-*positive subjects, 16 follow-up cases (53.3%) were included in this study, and follow-up periods were recorded from 1 to 23 years with average 9.75 ± 9.89 years. There was no hearing threshold increase during the short-term follow-up period (1-10 years), but the low-frequency and high-frequency hearing thresholds showed a significant increase with the prolongation of follow-up time. The speech discrimination score progressed gradually and significantly along with the course of the disease and showed a more serious decline, which was disproportionately worse than the pure tone threshold. In addition to the X-linked recessive inheritance pattern, the X-linked dominant inheritance pattern is also observed in *AIFM1*-related AN and affects females. In conclusion, we confirmed that *AIFM1* is the primary related gene among late-onset AN cases, and the most common recurrent variant is p.Leu344Phe. Except for the X-linked recessive inheritance pattern, the X-linked dominant inheritance pattern is another probability of *AIFM1*-related AN, with females affected. Phenotypical features of *AIFM1*-related AN suggested that auditory dyssynchrony progressively worsened over time.

## 1. Introduction

Auditory neuropathy (AN) is a special type of sensorineural hearing loss with a main manifestation of impaired speech comprehension, accounting for 1.2-10% of cases of hearing loss, depending on the population [1, 2]. The affected hearing in AN is mainly low frequency, and the speech recognition rate is obviously disproportionately lower than the pure tone threshold (PTA). This type of disease may arise from the inner hair cells (IHCs) of the cochlea, the synapses between the IHCs and the auditory nerve, the spiral ganglion neuron (SGN), the cochlear nerve fibers, and one or more of the auditory nerves [3].

The pathogenic mechanism of AN is currently unclear, and genetic factors may account for up to 40% of the pathogenesis of AN [4]. The inheritance pattern of AN includes autosomal recessive, autosomal dominant, and X-linked recessive inheritance. In 2006, our group located the gene locus AUNX1 of X-linked recessive hereditary neuropathy in the Xq23-q27.3 region for the first time [5] and then further identified *AIFM1* as the gene responsible for this kind of AN using whole exome sequencing technology in 2015 [6]. Apoptosis-inducing factor (AIF) is a flavin protein that is located in the mitochondrial membrane space. It was originally discovered as the first apoptotic factor that causes caspase-independent apoptosis [7]. This protein plays a critical role in maintaining the normal morphology and physiological functions of mitochondria and causing apoptosis that is not dependent on caspase.

Since AN was first identified more than 20 years ago, diagnosis, particularly precision diagnosis with lesion site identification, remains a challenge. Cases with genetic basis and the identification of the genes may be helpful for the lesion site identification, deciphering the underlying mechanism of AN [8–9]. Except for diagnosis, intervention is another challenge for clinical management for AN. Hearing aids and cochlear implantations, which are typical intervention strategies for cochlear sensory hearing loss, have variable outcomes for AN cases depending on the affected lesion sites [3–10]. Gene therapy may provide possibility for the treatment of AN [11]. Since virally mediated gene expressions in almost 100% HCs are possible, the treatment of presynaptic AN is possible [12]. And the virally expressing genes in SGNs are also feasible, supporting the possibility of treating postsynaptic AN [13].

Up to date, there is no frequency data of *AIFM1*-positive cases in AN cases. In this study, we further identified another 20 AN cases with *AIFM1* variants, including 7 novel variants and one hotspot variant, showing that the proportion of AN caused by *AIFM1* in Chinese patients with delayed-onset AN was as high as 18.6% (36/194), higher than the 15.53% (16/103) observed in the previous study [6]. Genotype-phenotype correlation analyses of AN cases with *AIFM1* gene pathogenic mutations were carried out, including the clinical hearing vestibular test, comprehensive clinical follow-up study data, and an in-depth exploration of the clinical characteristics of the disease and the related pathogenesis, to explore the characteristics of *AIMF1* gene-positive AN, laying the theoretical basis of AN classification diagnosis. The X-linked dominant inheritance pattern is also firstly observed in *AIFM1*-related AN and affects females in the study as well.

## 2. Materials and Methods

### 2.1. Ethics Statement

The study was approved by the Committee of Medical Ethics of Chinese PLA General Hospital. Written informed consent was obtained from all participants.

### 2.2. Subject Recruitment and Clinical Evaluation

A total of 50 patients with *AIFM1* mutations who were diagnosed with AN in the Chinese PLA Institute of Otolaryngology, Chinese PLA General Hospital, from April 1997 to June 2019 were recruited for this study. The diagnostic criteria were as follows: The typical audiological characteristics were that the auditory brainstem response (ABR) had no obvious differentiation waveform or severe abnormality and that the otoacoustic emission (OAE) and/or the cochlear microphonic (CM) potential could be normally extracted. Personal or family medical evidence of hearing loss, tinnitus, vestibular symptoms, and other clinical abnormalities of both the affected members and the unaffected members of these families was identified. Pure tone threshold (PTA), speech discrimination score (SDS), ABR, OAE, CM, and electrocochleography (ECochG) were carried out as otological examination batteries to evaluate auditory status. In general, the low frequencies were primarily affected; thus, we focused on the low-frequency data and calculated PTA as the average of the thresholds of 250-1000 Hz to avoid bias in the assessment of the degree of AN hearing loss. Vestibular function evaluation included vestibular evoked myogenic potentials, oculomotor function tests, positional nystagmus tests, positioning nystagmus tests, and bithermal caloric tests. High-resolution computed tomography (CT) scans of the temporal bone and cerebral magnetic resonance imaging (MRI) were performed to exclude other possible neuropathic or anatomical disorders.

### 2.3. Genetic Techniques

Next-generation sequencing and Sanger sequencing were performed on the patients as previously described. Variation interpretation (evaluation of the pathogenicity) was based on the standards and guidelines of the American College of Medical Genetics and Genomics and the Association for Molecular Pathology (ACMG and AMP) [14].

### 2.4. Statistical Analysis

Statistical analysis was performed using SPSS 19.0 statistical software, Empower software (http://www.empowerstats.com, X&Y Solutions, Inc., Boston, MA) and R software (https://www.R-project.org), as well as G. Comparisons of the two sets of data were performed using an independent sample *t*-test. The comparisons of multiple sets of data were performed using one-way ANOVA. *p* < 0.05 represented a significant difference. Spline smoothing was performed using GAMM (generalized additive mixed model) to explore the change in pure tone threshold with the length of follow-up time.

## 3. Results

### 3.1. General Clinical Information

Fifty patients with *AIFM1* mutations were recruited for this study, including 30 patients who have been previously studied and 20 novel patients who were identified recently (Table 1). All patients had no history of high-risk factors such as hyperbilirubinemia and hypoxia and denied a history of metabolic diseases such as diabetes. Nine (18%) patients had a history of ototoxic drug use, and one patient had a history of exposure to noise. Clinically, 37 patients (74%) complained of tinnitus at the first visit, and numbness of the extremities was the second most common symptom (12 patients), while few patients experienced visual impairment (10 patients) and vertigo (8 patients). Fifteen patients (30 ears) received cVEMP examination, among which 19 (63.3%) ears showed ipsilateral sacculus dysfunction, and 11 (36.7%) ears showed normal function. Nineteen patients underwent vestibular function tests; 15 sides (15/38, 39.5%) showed abnormalities, while the other 23 sides were normal. No abnormalities in temporal CT were found, and 3 patients (42.9%, 3/7) showed bilateral cochlear nerve hypoplasia in the MRI test.

**Figure 1:**
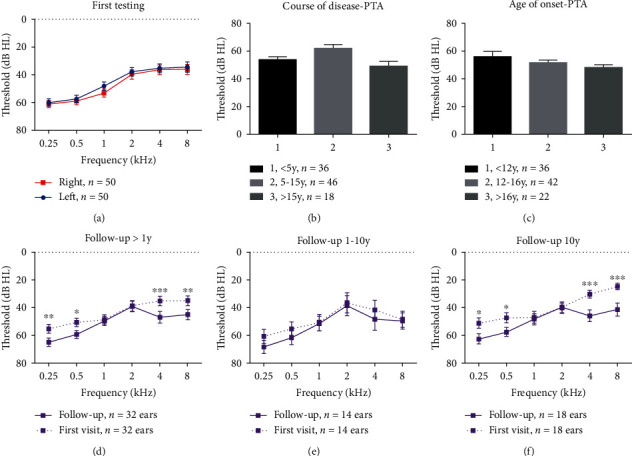
Pure tone test of the *AIFM1*-positive cases. (a) Average threshold of the 50 cases with *AIFM1* variations. (b) Mean PTA in the groups with different disease courses. (c) Mean PTA in the groups with different onset ages. (d-f) Mean threshold in each frequency with different follow-up periods. dB: decibels; Hz: Hertz; PTA: pure tone average.

The pure tone thresholds of each frequency were not significantly different between the left and right ears in all 50 patients (Figure 1(a)). The disease durations at the first visit ranged from 0 to 33 years (9.7 ± 7.7 years), and the PTAs among the 5-, 5- to 15-, and >15-year disease duration groups were not significantly different (*p* > 0.05) (Figure 1(b)). The onset ages of *AIFM1*-positive patients ranged from 5 to 20 years (13.4 ± 3.9). In addition, the earlier the onset, the more severe the hearing impairment. The pure tone averages (PTAs, 250-1000 Hz) of the <12-year-old group, 12- to 16-year-old group, and >16-year-old group were 55.7, 51.2, and 47.7 dB HL, respectively. The most severe hearing loss was observed in the youngest age group. However, the differences in hearing loss between the three onset age groups were not statistically significant (Figure 1(c)).

### 3.2. Mutation Spectrum of *AIFM1* (Table 2)

Among the 20 novel cases found with *AIFM1* variations, seven novel and three reported variations were found, located in the FAD, NADH, and C-terminus. Pathogenicity was assessed using SIFT (http://sift.jcvi.org/), PolyPhen-2 (Polymorphism Phenotyping V.2, http://genetics.bwh.harvard.edu/pph2), LRT (http://www.genetics.wustl.edu/jflab/lrt_query.html), and MutationTaster (http://www.mutationtaster.org).

The seven novel variations were all pathogenic with the evidences of “PS×1 + PM×2 + PP×2” according to the ACMG and AMP guidelines [14]. Firstly, 18.6% of late-onset AN cases had variants in *AIFM1*, while the variations were absent in our sensorineural hearing loss group (PS4). Secondly, these variations were absent or at extremely low frequency in ESP, ExAC, gnomAD-EAS, or 1000genomes (PM2). Thirdly, in these families, the variations were cosegregated with AN phenotype, with proband's mother carrying variation but having no AN performance (PM). Fourthly, these variants were predicted to be deleterious with SIFT, PolyPhen-2, LRT, MutationTaster, and so on (PP3). In addition, in terms of phenotype, these patients were all reported as AN(PP4). Furthermore, cells with the *AIFM1* mutation led to decreased dimerization and impaired mitochondrial functions (unpublished data), which may indicate that the mutations in *AIFM1* gene may affect auditory function, providing PS3 evidence.

The recurrent variants were p.Ile304Met, p.Leu344Phe, p.Arg422Trp, and p.Tyr560His, among which the most common variant in the AN population was p.Leu344Phe, which was present in 36.1% (13/36) of the positive cases, followed by p.Arg422Trp (13.9%, 5/36).

**Figure 2:**
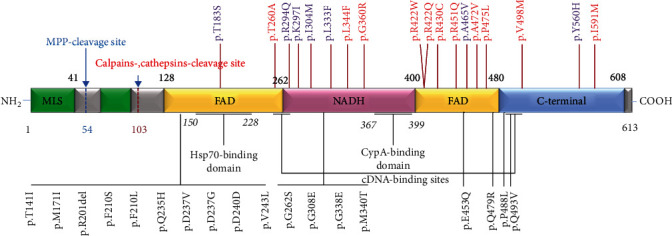
*AIFM1* variations in auditory neuropathy as well as other syndromes. The purple ones are the new variations identified in this study, the red ones are the previously reported AN-related variations, and the black ones are the variants that are related to syndromes such as cerebellar ataxia (modified from [6]).

In total, 18 variations in *AIFM1* variations were related to the AN phenotype, with 9 variations located in FAD, 6 variations in NADH, and 4 variations in the C-terminal region. There was no overlap with the other *AIFM1* variations that caused other syndromes [6, 15–28] (Figure 2).

### 3.3. Genotype and Phenotype Correlation Analysis of the 20 Newly Identified Cases

#### 3.3.1. Clinical Features of the 20 Newly Identified Cases with *AIFM1* Variants (Table 1)

Except for families 0804755 and 1507328, who had a family history, 18 other cases with *AIFM1* variants were sporadic cases. The age of onset ranged from 6 to 20 years, with only one case (1507426) not complaining of childhood-onset AN. Except for one female case with unilateral AN, all other patients showed bilateral AN. The audiograms varied, with 31 ears (83.8%, 31/37) showing upsloping types. Seven of 20 patients underwent inclined sagittal MRI of the internal auditory canals, with 3 patients showing bilateral cochlear nerve hypoplasia.

#### 3.3.2. Female Patients with *AIFM1* Variants (Possible X-Linked Dominant Inheritance Pattern)

**Figure 3:**
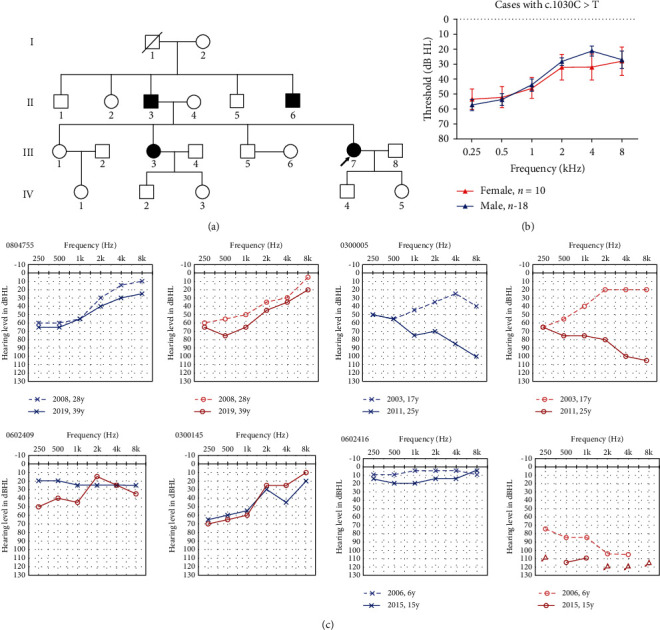
Family trees and audiological characteristics of the five female cases. (a) Family trees of the family 0804755. (b) Mean hearing threshold of the cases with the *AIFM1* c.1030C>T (p.Leu344Phe) variation. (c) Audiograms of the five female affected cases. Symbols “o” and “x” denote air conduction pure tone thresholds at different frequencies in the right and left ears. dB: decibels; Hz: Hertz. The dashed line represents the audiograms detected in the first time, while the solid lines were the latest audiological examinations. y: years old.

All of the female cases had the same variant, c.1030C>T (p.Leu344Phe), which was also the most common variant among AN-related *AIFM1* variations. Except for 0804755 (Figure 3(a)), the other female patients had no family history. There was no hearing threshold difference between male and female AN patients with this variant (Figure 3(b)). However, the audiograms of the females affected varied from normal to profound, including flat, upflopping, and downflopping as time went on (Figure 3(c)).

#### 3.3.3. Phenotype Follow-Up of the 30 AN Patients with *AIFM1* Mutations

**Figure 4:**
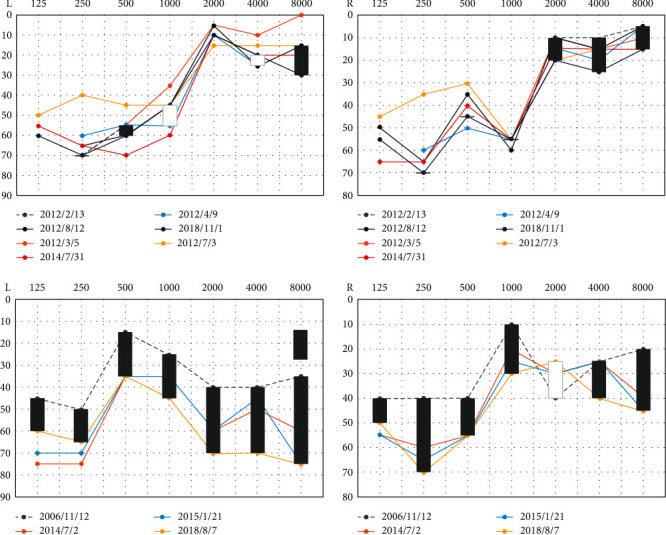
Typical cases with stable and progressive pure tone hearing thresholds.

Sixteen patients (53.33%) underwent follow-up pure tone audiometry tests, and the follow-up period was 1-23 years, with a mean time of 9.75 ± 9.89 years. The hearing threshold change varied between patients (Figure 4). The average thresholds of low and high frequencies in the different follow-up groups were significantly different in all of the patients who had been followed for more than one year (Figure 1(d)). For the patients with a short-term follow-up period (1-10 years), the hearing deterioration was not apparent at all frequencies (Figure 1(e)), but with the prolongation of follow-up time (more than 10 years), the low-frequency (0.25 and 0.5 kHz) and high-frequency (4 and 8 kHz) hearing thresholds showed a significant increase (Figure 1(f)).

**Figure 5:**
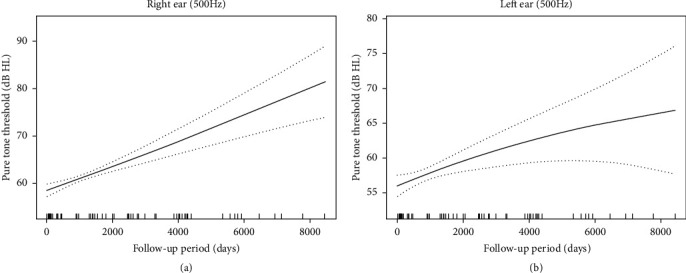
(a, b) The change of pure tone threshold of 0.5 kHz over time in both ears of *AIFM1*-positive AN patients. Spline smoothing was performed using GAMM (generalized additive mixed model) to explore the change of pure tone threshold with the length of follow-up time. The solid lines represent the fitting spline. The dashed lines represent the 95% confidence intervals. The vertical axis measures the change in pure tone hearing. The rug plot provides a visual representation of the frequency distribution for follow-up time. Each individual data point is represented by a single tick mark at the appropriate location on the chosen time scale (days).

**Figure 6:**
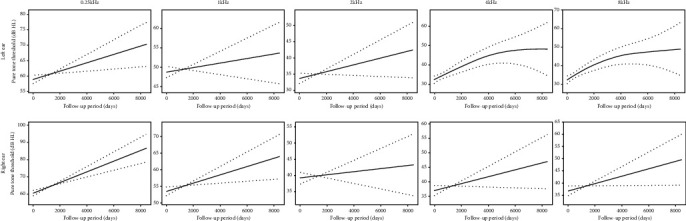
The change of pure tone threshold of 0.25, 1, 2, 4, and 8 kHz over time in both ears of *AIFM1*-positive AN patients. Spline smoothing was performed using GAMM (generalized additive mixed model) to explore the change of pure tone threshold with the length of follow-up time. The solid lines represent the fitting spline. The dashed lines represent the 95% confidence intervals.

In addition, we performed spline smoothing by using GAMM to explore the change in the pure tone threshold with the length of follow-up time.  Figure 5 illustrates the shape of the relationship between the hearing outcome at a frequency of 0.5 kHz and the follow-up time (edf = 1.427, *p* = 0.0096). This result suggested that the hearing threshold of 0.5 kHz in both ears worsened gradually over time. The hearing thresholds of 0.25, 1, 2, 4, and 8 kHz changed in a similar pattern as that of 0.5 kHz, although statistical significance was not reached in either ear (Figure 6).

Twenty-three patients (46/60 ears, 76.67%) with *AIFM1* mutations underwent binaural speech testing, and 10 patients (20 ears, 43.5%) were followed up. A total of 10 patients (20 ears) were followed up for SDS (Table S1), with a follow-up period of 1-15 years (6.80 ± 4.47 years). Of the 46 ears, 50% of patients with mild hearing loss had zero SDS, which was a much higher figure than that in the moderate and severe groups. Overall, the SDS of the mild hearing loss group was significantly lower than that of the moderate and severe groups (*p* < 0.05). Therefore, the degree of SDS decline in these patients with AN was not proportional to the pure tone hearing threshold, and SDS was more severe in patients with mild hearing loss than in patients in the moderate and severe hearing loss groups (Table S2). Disease duration was another risk factor; of the 9 patients with a disease duration less than 5 years, none had an SDS score of zero. In patients with disease duration of more than 5 years, the proportion of patients with an SDS score of zero increased significantly, accounting for 50% of all ears (Table S3). This finding indicates that SDS decreased significantly with the prolongation of the disease course, and the difference was statistically significant (*p* < 0.05). The mean values of the left and right ears before and after follow-up were greater than zero, indicating that the SDS exhibited a downward trend before and after follow-up, but the difference between the two ears was not statistically significant (*p* > 0.05).

Among the 30 patients, 7 ears from 5 patients were able to elicit V-waves, but the waveform differentiation was poor, the amplitudes were reduced, and the latencies were prolonged. The PTAs of the 7 ears with the V-wave were relatively better, and the course of the disease was shorter than that of the unexposed ABR waveform, but the difference was not statistically significant (*p* > 0.05) (Table S4). Three of five patients had follow-up ABR data; their V-wave latencies were gradually extended, and the V-wave of ABR was unextracted in the follow-up of one ear (left ear of 1007170-1) (Table S5).

All 30 patients underwent DPOAE; 29 patients passed with the elicitation of at least five frequencies, while one patient had no response at any of the frequencies. This individual was a member of family 1007170 and had a disease duration of 33 years. AN may progress to sensorineural hearing loss, with outer hair cell impairment as time goes on.

A total of 22 patients (44 ears) underwent electrocochleography examination, among whom, 6 patients underwent follow-up observation. The -SP waves were found in 21 patients, except for one person who had an unobvious wave. Nine patients (20.5%, 9/44) showed the -SP wave only, without an obvious CAP wave, while the remaining 35 ears showed both -SP and CAP waveforms, with absolute values of ‐SP/AP > 0.4 (Table S6). The degree of hearing loss in patients with AN who did not elicit CAP waveforms was significantly higher than that in patients with CAP, and the difference was statistically significant (*p* < 0.001). For the 6 cases (12 ears) with a follow-up ECochG test (Figure S1), there were no differences between the absolute values of SP/AP.

## 4. Discussion

AN is a special type of hearing dysfunction disease, which is one of the critical diseases that cause speech communication disorders in infants and adolescents [3]. In the auditory system, HCs and SGNs are very important for hearing ability; HCs convert the sound waves into electrical signals, and SGN transmit the electrical signals into the auditory cortex for hearing ability [29]. In a mammal's cochlea, HCs and SGNs are vulnerable for multiple damages, including noise, gene mutation, ototoxic drugs, inflammation, and aging [30–34]; while the mammal's cochlea only have very limited HC and SGN regeneration ability, majority of the damaged HC and SGN cannot be spontaneously regenerated [35–41]. Thus, hearing loss is usually irreversible, and AN may come from the damage of IHC and SGNs.

As a difficult and popular topic in international research, research on AN has been performed for 20 years, from preliminary reports to various explorations of its pathogenesis, and it is beginning to be gradually understood accurately. The etiology of AN varies with age, genetic factors, hyperbilirubinemia, low birth weight, premature birth, and hypoxia. More patients may be discovered as the use of genetic testing in the diagnosis of auditory neuropathy becomes more widespread [4–8]; however, no prevalence studies have been performed to date. In our previous study, we confirmed that *OTOF* is the most common gene-causing congenital auditory neuropathy [42]. In contrast, for patients with late-onset AN, the etiology varies and is associated with optic atrophy, sensorimotor neuropathy, and other peripheral neuropathies. Among the late-onset cases, *AIFM1* is reported to be the most common genetic cause [6]. In this study, we further confirmed that *AIFM1* is the most common genetic cause of all noninfant-onset AN cases. The identification of genes is helpful to identify related lesion sites of AN and contributes to a better understanding of the underlying pathogenic mechanisms [8, 9].

*The AIFM1* gene, also known as AIF, PDCD8, COXPD6, etc., is located in the human chromosome Xq25-q26 region, with a full length of 36.471 kb and 16 exons encoding a full-length 613 amino acid protein. In the mitochondria, *AIFM1* acts as a FAD-dependent NADH oxidoreductase and plays a critical physiological role in the stable and mature mitochondrial oxidative respiratory chain complex I and the elimination of peroxide. In this study, we further expanded the mutation spectrum and long-term phenotypes of *AIFM1*-related cases. We found another 20 AN cases with *AIFM1* variants by whole genome sequencing and Sanger sequencing, including 7 novel variants. All 18 AN-related variations had no overlap with the other phenotype-related *AIFM1* variations (Table S7). In addition, we confirmed that the most common variation is *AIFM1* c.1030C>T (p.Leu344Phe). Due to its location in the loop region, this variation may have an influence on folding.

In this study, we found 5 female AN cases with *AIFM1* variants, the phenotype of whom was similar to those of the male cases with the same variant. *AIFM1* gene mutation AN may also be inherited in an X-linked dominant inheritance pattern. In our previous study, except for the patients undergoing whole exome sequencing, we did not pay attention to female AN cases; only male cases were tested for the *AIFM1* gene.

To decipher the phenotype progression of *AIFM1*-related AN, a follow-up study was performed. Further genotype-phenotype correlation analysis can effectively help physicians and patients understand the disease-causing mechanism, process, and outcome of disease by research methods. To further assist in the consultation and evaluation of prognosis in patients with clinical AN, the clinical phenotypes of *AIFM1*-related cases were as follows:

1. For the pure tone threshold, AN patients have large individual differences in audiologic phenotype. Although the ascending audiogram is the typical audiometric pattern of AN patients, audiograms with various configurations and varying severities may occur. The hearing of some patients may improve, while others' hearing loss may remain stable for a long time or even worsen. Among the very limited reports involving the follow-up characteristics of AN patients, the hearing outcomes in a long-term follow-up remain elusive [43]. Not surprisingly, we found, in the 50 *AIFM1*-positive cases, that the low frequency spectra were mostly affected, especially in the 0.25-1 kHz range. Hearing impairment ranged from mild to moderate. No significant differences were detected in the hearing thresholds tested between the first and final visits within 1-10 years of follow-up. However, when the follow-up periods were prolonged to over ten years, hearing thresholds in both the low frequencies (0.25-0.5 kHz) and the high frequencies (4-8 kHz) showed significant worsening. In addition, the pure tone threshold tended to deteriorate over time, especially at a frequency of 500 Hz
2. For SDS, the AN patient's prominent complaint is that he/she can hear voices without understanding the meaning. Our study found that patients with mild hearing loss had a high proportion of a score of zero when testing SDS, which was much more severe than the SDS in the moderate and severe hearing loss groups, and the degree of SDS was not consistent with the pure tone threshold. This fact suggests that in clinical audiology assessment, the SDS performance of AN patients is much more critical than that of PTA. The evaluation of the degree of hearing loss by PTA alone may underestimate the patient's condition. Furthermore, we followed up on the SDS of this type of AN patient and found that SDS decreased significantly with the prolongation of the disease course. This result indicates that the neurological synchronization of the *AIMF1* gene-related AN is gradually aggravated
3. ECochG revealed that -SP and CAP waves existed together in 80% of cases, but the SP/AP values were higher than normal. Patients showed worse hearing loss when their CAP waves disappeared. Compared with DPOAE, cochlear electrograms can help us locate the lesions of AN [44]. The -SP wave reflects the fractional depolarization process after the inner hair cells are subjected to the acoustic signal and is the maximum amplitude recorded by the needle electrode placed on the cochlear or round window through the tympanic membrane [45]. The -SP waves are mainly derived from inner hair cells, and their amplitude and latency are objective indicators of the function of inner hair cells. Except for one patient without obvious one-time single-SP, the -SP wave of all of the other AN patients could be seen, suggesting that the AN lesion caused by *AIFM1* gene mutation may be located in the auditory conduction pathway outside the inner hair cells. CAP is produced in cochlear spiral ganglion cells and is an afferent nerve response. The decrease in CAP amplitude can prove the synchrony decline in auditory nerve activity. We observed that the -SP and CAP waveforms were present in 80% of patients at the same time, but the absolute value of -SP/AP was >0.4, which was higher than normal, suggesting that there was a loss of synchronization of auditory nerve activity. This also explains why the SDS decline in this type of patient is more marked than the decrease in PTA from the perspective of physiology and pathology. Furthermore, we analyzed the patient's auditory condition based on the presence or absence of CAP waves, showing that the degree of hearing loss without CAP waveform was more serious. Clinically, the detection value should be increased, and the degree of hearing loss should be comprehensively judged

Individuals with lesions affecting the auditory nerve showed poor performance with cochlear implantation, since the neural transmission was affected [2]. The audiological phenotype features of *AIFM1*-related AN suggested that auditory dyssynchrony progressively worsened over time. Electrophysiological examinations of the cochlear nerve indicated that lesions would be located on the auditory pathway from postsynapses to acoustic fibers [3]. Further, the diffusion-weighted MRI (dMRI) analysis techniques may contribute to the microstructure of the auditory tracts in vivo in individuals with AN [3, 46]; from the dMRI of some patients, we can see a reduction in apparent fiber density within the auditory brainstem tracts, which is consistent with the assumed pathophysiological mechanism of postsynapses to acoustic fibers (unpublished data). Thus, AN patients with *AIFM1* mutation may have poor efficiency from cochlear implantation.

The molecular disease-causing mechanism of *AIFM1* mutation-related hearing loss is still unclear; the mitochondrial function and the caspase-independent apoptosis to neuronal development and adult neurogenesis play a critical role in previous studies [47, 48]. In our recent study, we found that the *AIFM1* mutation led to decreased dimerization and further impaired mitochondrial functions, such as increase of ROS production and impairment of mitochondrial membrane potential, thereby activating caspase-independent apoptosis (unpublished data). However, the complete and clear pathogenesis of AN and the genotype-phenotype correlation need to be further clarified.

In conclusion, (1) *AIFM1* is the most common genetic cause of late-onset AN, with the hotspot mutation of c.1030C>T (p.Leu344Phe). (2) In addition to the X-linked recessive inheritance pattern, the X-linked dominant inheritance pattern is another probability of *AIFM1*-related AN, which affects females. (3) The hearing threshold of AN patients with *AIFM1* mutation tends to worsen when the follow-up period is prolonged. Phenotype features of *AIFM1*-related AN suggested that auditory dyssynchrony progressively worsened over time. Electrophysiological examinations of the cochlear nerve indicated that lesions would be located on the auditory pathway from postsynapses to acoustic fibers.

## Figures and Tables

**Table 1:** Clinical phenotype of the 20 *AIFM1*-positive auditory neuropathy cases.

Number	Case ID	Gender^a^	Age of test (years)	Hearing impairment phenotype			PB max (%)^c^	Tinnitus	Vertigo	Gait instability	Numbness of extremities	Visual impairment	Muscle deformity	Intellectual abilities	MRI of brain^**d**^
				Age of onset (years)	Hearing loss degree (PTA) (L/R)^b^	Type of audiometry									
1	300005	F	17	15	Both mild	Upsloping	NA	+	−	NA	NA	Myopia	NA	−	NA
2	300145	F	21	18	Both moderate	Upsloping	NA	+	−	NA	NA	−	NA	−	−
3	300158	M	14	10	Moderate/moderately severe	Upsloping	NA	−	−	−	−	−	−	−	NA
4	400352	M	20	16	Moderate/moderately severe	Upsloping	NA	NA	NA	NA	NA	Myopia	NA	−	NA
5	400469	M	21	14	Severe/moderate	Other	NA	−	−	NA	NA	−	NA	−	CNH
6	400738	M	28	20	Both moderate	Upsloping	28/24	+	NA	+	+	NA	NA	−	−
7	501418	M	21	12	Both mild	Upsloping	50/84	+	−	−	−	−	−	−	NA
8	602409	F	17	13	Both mild	Flat/upsloping	NA	−	−	−	−	−	−	−	NA
9	602416	F	6	6	Normal/severe	Flat/downsloping	96/NA	+	−	+	+	−	NA	−	NA
10	703306	M	27	12	Severe/moderately severe	Upsloping	NA	+	+	+	+	−	NA	−	NA
11	804755	F	28	21	Mild/moderate	Upsloping	24/32	+	−	NA	NA	Myopia	NA	−	CNH
12	1007198	M	20	10	Both moderately severe	Upsloping	0/0	+	+	−	−	−	−	−	NA
13	1507328	M	28	15	Moderately severe/moderate	Upsloping	0/0	+	−	NA	NA	−	NA	−	NA
14	1507329	M	24	10	Both moderately severe	Upsloping	36/40	−	−	NA	NA	−	NA	−	−
15	1507366	M	20	8	Normal/mild	Upsloping/flat	NA	−	−	−	−	NA	−	−	NA
16	1507405	M	34	17	Both moderate	Upsloping	56/52	+	−	NA	NA	−	NA	−	NA
17	1507426	M	25	20	Moderate/normal	Upsloping/flat	0/68	+	−	NA	NA	NA	NA	−	−
18	1707671	M	18	14	Both mild	Upsloping	8/60	−	−	NA	NA	Myopia	NA	−	NA
19	1707676	M	21	14	Both mild	Upsloping	88/96	+	+	NA	NA	Myopia	NA	−	NA
20	1707834	M	16	11	Both mild	Upsloping	56/28	+	−	NA	−	Myopia	NA	−	CNH

Note: ^a^F: female; M: male. ^b^PTA: pure tone average; L: left; R: right. ^c^CNH: cochlear nerve hypoplasia. NA: not available; +/−: positive or negative finding.

**Table 2:** Variations identified in the 20 *AIFM1*-positive cases.

Nucleotide change	Amino acid change	Protein domain	Number of patients	Reported	Prediction information				Minor allele frequency^c^			
					SIFT^a^	PolyPhen-2-HVAR^b^	LRT	MutationTaster	ESP	ExAC	gnomAD-EAS	1000genomes
c.547A>T	p.Thr183Ser	FAD	1	No	0.05	0.228	Deleterious	Disease-causing	-1	-1	NA	-1
c.881G>A	p.Arg294Gln	NADH	1	No	0.63	0.148	Deleterious	Disease-causing	-1	5.70399*E*-05	-1	-1
c.890A>T	p.Lys297Ile	NADH	1	No	0.01	0.788	Deleterious	Disease-causing	-1	-1	0.00291262	0.0005
c.912C>G	p.Ile304Met	NADH	2	No	0.11	0.846	Deleterious	Disease-causing	-1	-1	-1	NA
c.997C>T	p.Leu333Phe	NADH	1	No	0.11	0.846	Deleterious	Disease-causing	-1	NA	NA	NA
c.1030C>T	p.Leu344Phe	NADH	8	Yes	0.15	0.457	Deleterious	Disease-causing	-1	0.000205173	0.00291262	0.0005
c.1264C>T	p.Arg422Trp	FAD	2	Yes	0.09	0.999	Deleterious	Disease-causing	-1	-1	NA	-1
c.1394C>T	p.Ala465Val	FAD	1	No	0.002	1	Deleterious	Disease-causing	-1	NA	NA	NA
c.1492G>A	p.Val498Met	C-terminal	1	Yes	0.02	0.991	Deleterious	Disease-causing	1.13947*E*-05	-1	0.0000777001	-1
c.1678T>C	p.Tyr560His	C-terminal	2	No	0.01	0.9	Deleterious	Disease-causing	-1	NA	NA	NA

Note: ^a^deleterious (≤0.05); tolerance > 0.05). ^b^Probably damaging (≥0.957), possibly damaging (0.447 ≤ pp2_havr ≤ 0.909), and benign (≤0.446). ^c^Allele frequencies in each population database; it is marked as “-1” when the allele is not carried in the corresponding group. EAS: East Asians. NA: not available.

## Data Availability

The datasets used and/or analyzed during the current study are available from the corresponding author on reasonable request.

## Supplementary Materials

Supplementary 1 Figure S1: follow-up ECochG test of 6 cases (12 ears); there was no difference between the absolute values of -SP/AP.F

Supplementary 2 Table S1: difference value of SDS in *AIFM1*-positive cases. Table S2: correlation of PTA and SDS. Table S3: SDS from cases with different disease courses. Table S4: the PTA and disease course of the cases with ABR V-wave. Table S5: follow-up of the cases with ABR V-wave. Table S6: CAP waves and PTA in *AIFM1*-positive AN cases. Table S7: the reported variations and diseases of *AIFM1.*
