# Supplementary material for: High Frequency of AIFM1 Variants and Phenotype Progression of Auditory Neuropathy in a Chinese Population
Source: Neural Plast. 2020 Jul 1;2020:5625768. doi: 10.1155/2020/5625768 (PMC7350177; doi:10.1155/2020/5625768)
Supplement: Supplementary 1 — Figure S1: follow-up ECochG test of 6 cases (12 ears); there was no difference between the absolute values of -SP/AP.F [file 5625768.f1.docx]

Table S1 Difference value of SDS in AIFM1 positive cases

| ID | SDS-L  (difference value) | SDS-R  (difference value) | Follow-up period (years) |
| --- | --- | --- | --- |
| 1007170-1 | 8 | 4 | 1 |
| 0501806 | 4 | 0 | 1 |
| 0702724-2 | -8 | -12 | 2 |
| 0702724-1 | 4 | 12 | 7 |
| 0703305 | 36 | 24 | 7 |
| 0602423-5 | 16 | -8 | 8 |
| 0602423-1 | -4 | 0 | 8 |
| 0602423-2 | 8 | -4 | 8 |
| 0400223-2 | -2 | 12 | 11 |
| 0400223 | 34 | -12 | 15 |
| Average | 9.60±15.02 | 1.60±11.65 | 6.80±4.47 |

Table S2 Correlation of PTA and SDS

| PTA (250-1000Hz) | Ears | SDS=0 (ears) | SDS>0 (ears) | SDS (%) |
| --- | --- | --- | --- | --- |
| 26－40dB HL | 8 | 4 | 4 | 13.75±18.59 |
| 41－60dB HL | 22 | 3 | 19 | 41.91±30.29 |
| 61－80dB HL | 13 | 5 | 8 | 31.69±33.00 |
| >80 dB HL | 3 | 2 | 1 | 1.33±30.70 |

Table S3 SDS from cases with different disease courses

| Disease duration(years) | Ears | SDS=0 (ears) | SDS>0 (ears) | SDS(%) |
| --- | --- | --- | --- | --- |
| <5 | 18 | 0 | 18 | 47.22±26.43 |
| 5-15 | 12 | 6 | 6 | 21.50±33.39 |
| >15 | 16 | 8 | 8 | 21.25±27.00 |

Table S4 The PTA and disease course of the cases with ABR V-wave

|  | PTA（250-1000Hz） | Course of disease (years) |
| --- | --- | --- |
| V wave(7ears） | 49.76±18.06 | 6.30±9.00 |
| NR (47 ears) | 57.16±21.12 | 11.77±8.40 |

Table S5 Follow-up of the cases with ABR V-wave

| Time of testing | Cases | ABR-L | ABR-R |
| --- | --- | --- | --- |
| 2002/12/12 | 0400223-1 | V=5.68ms | NR |
| 2013/7/18 | 0400223-1 | V=6.20ms | NR |
| 2005/8/30 | 0501757 | V=6.15 | V=6.25 |
| 2006/1/11 | 0501757 | V=6.35 | V=6.35 |
| 2013/8/20 | 1007170-1 | V=6.33ms | NR |
| 2014/7/17 | 1007170-1 | NR | NR |

NR: No response

Table S6 CAP waves and PTA in AIFM1 positive AN cases

|  | Ears | PTA (250-1000Hz) |
| --- | --- | --- |
| No CAP | 9 | 70.74±14.66. |
| With CAP | 35 | 56.10±17.40 |

Table S7. The reported variations and diseases of *AIFM1*

| **Year** | **Country** | **Author** | **Variation** | **Diseases** |
| --- | --- | --- | --- | --- |
| 2018 | Chinese | Wang Binghao | c.513G>A, p.M171I | Charcot-Marie-Tooth disease type 4, CMTX4 |
| 2017 | Spain | Paula Sancho | c.629T>C, p.F210S | Inherited peripheral neuropathies, IPNs |
| 2017 | Israel | Gali Heimer | c.1019T>C, p.M340T  c.422C>T, p.T141I | X-linked childhood cerebellar ataxia |
| 2017 | America | Hu Bo | c.630C>G, p.F210L | Late-onset axonal polyneuropathy |
| 2017 | multicenter | Noriko Miyake | c.710A>G, p.D237G  c.710A>T, p. D237V  c.705G>C, p.Q235H  c.720C>T, p.D240D | X-linked hypomyelination with spondylometaphyseal dysplasia(H-SMD) |
| 2017 | America | Sarah U. Morton | c.1436A>G, p.Q479R | Fatal encephalomyopathy and mitochondrial disease in an infant |
| 2016 | Poland | Mierzewska, Hanna | c.710A>G, p.D237G | Spondyloepimetaphyseal dysplasia with neurodegeneration |
| 2015 | Italy | Daria Diodat | c.1013G>A, p.G338E | Infantile motor neuron disease |
| 2015 | Germany | Matthias Kettwig | c.727G>T, p.V243L | Severe muscular atrophy |
| 2015 | Italy | Anna Ardisson | c.784G>A, p.G262S | Slowly progressive mitochondrial encephalomyopathy |
| 2012 | America | Rinaldi Carlo | c.1478A>T, p.E493V | Cowchock Syndrome (CMYX4） |
| 2011 | Israel | Itai Berger | c.923G>A, p.G308E | Early prenatal ventriculomegaly |
| 2010 | Italy | Daniele Ghezzi | c.601–603 deletion，R201 Del | Severe X-Linked Mitochondrial Encephalomyopathy |
