# Supplementary figures and images for: High Frequency of AIFM1 Variants and Phenotype Progression of Auditory Neuropathy in a Chinese Population
Source: Neural Plast. 2020 Jul 1;2020:5625768. doi: 10.1155/2020/5625768 (PMC7350177; doi:10.1155/2020/5625768)

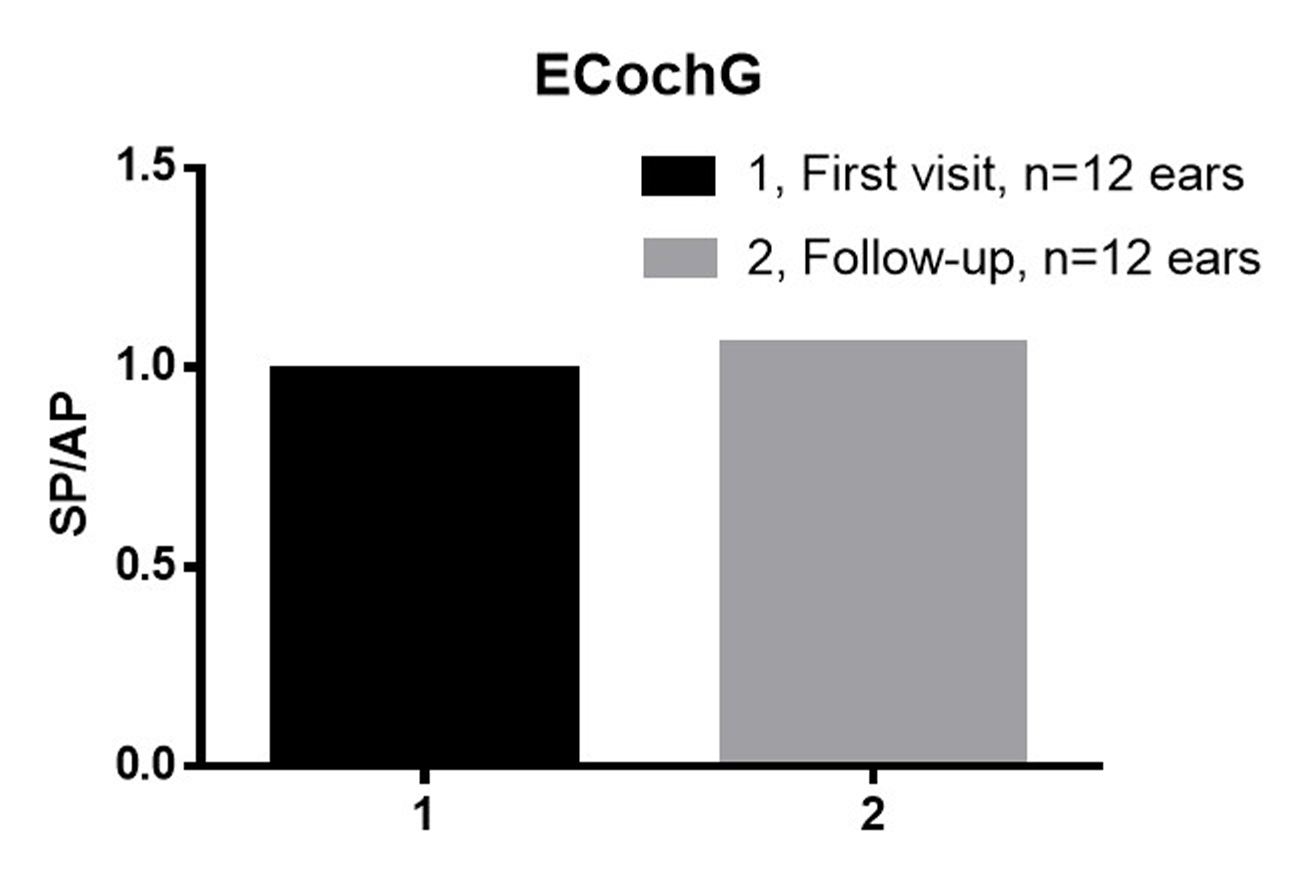

Supplement: Supplementary 2 — Table S1: difference value of SDS in AIFM1-positive cases. Table S2: correlation of PTA and SDS. Table S3: SDS from cases with different disease courses. Table S4: the PTA and disease course of the cases with ABR V-wave. Table S5: follow-up of the cases with ABR V-wave. Table S6: CAP waves and PTA in AIFM1-positive AN cases. Table S7: the reported variations and diseases of AIFM1. [file 5625768.f2.tif]
